# Author Correction: Engineering the formation of spin-defects from first principles

**DOI:** 10.1038/s41467-024-49945-z

**Published:** 2024-07-02

**Authors:** Cunzhi Zhang, Francois Gygi, Giulia Galli

**Affiliations:** 1 Pritzker School of Molecular Engineering, University of Chicago, Chicago, IL USA; 2 Department of Computer Science, University of California Davis, Davis, CA USA; 3 Department of Chemistry, University of Chicago, Chicago, IL USA; 4 Materials Science Division and Center for Molecular Engineering, Argonne National Laboratory, Lemont, IL USA

**Keywords:** Atomistic models, Electronic devices, Quantum information

Correction to: *Nature Communications* 10.1038/s41467-023-41632-9, published online 26 September 2023

The original version of this Article omitted relevant details in the Acknowledgements. The incorrect version of the Acknowledgements text read:

“We thank Yu Jin, Elizabeth M.Y. Lee, and Marco Govoni for useful discussions. This work was supported by MICCoM, as part of the Computational Materials Sciences Program funded by the U.S. Department of Energy, Office of Science, Basic Energy Sciences, Materials Sciences, and Engineering Division through Argonne National Laboratory. This work was also supported by the QNEXT hub (1F-60579). We acknowledge the computational resources at the University of Chicago’s Research Computing Center and the Argonne Leadership Computing Facility.”

The correct version of the Acknowledgements text should read:

“We thank Yu Jin, Elizabeth M.Y. Lee, Joe Heremans and Marco Govoni for useful discussions.The design, implementation and execution of the computational strategy adopted in this work, as well as two of the main codes used in this work, Qbox and SSAGES, were supported by MICCoM, as part of the Computational Materials Sciences Program funded by the U.S. Department of Energy, Office of Science, Basic Energy Sciences, Materials Sciences, and Engineering Division through Argonne National Laboratory. The extensive analysis of experimental data that led to the choice of materials and experimental conditions was supported by QNEXT. This research used computational resources at the University of Chicago’s Research Computing Center and the Argonne Leadership Computing Facility located at Argonne National Laboratory. Computer time at Argonne Leadership Computing Facility was provided by the Department of Energy’s ASCR Leadership Computing Challenge (ALCC).”

This has now been corrected in both the PDF and HTML versions of the Article.

