# Supplementary material for: Engineering the formation of spin-defects from first principles
Source: Nat Commun. 2023 Sep 26;14:5985. doi: 10.1038/s41467-023-41632-9 (PMC10522650; doi:10.1038/s41467-023-41632-9)
Supplement: Supplementary file 1 — Supplementary Information [file 41467_2023_41632_MOESM1_ESM.pdf]

Supplementary Information for:
Engineering the formation of spin-defects from first principles

Cunzhi Zhang,[1] Francois Gygi,[2] and Giulia Galli[1,3,4,*]

[1] *Pritzker School of Molecular Engineering, University of Chicago, Chicago, IL 60637, USA*

[2] *Department of Computer Science, University of California Davis, Davis, CA 95616, USA*

[3] *Department of Chemistry, University of Chicago, Chicago, IL 60637, USA*

[4] *Materials Science Division and Center for Molecular Engineering, Argonne National Laboratory, Lemont, IL 60439, USA*

[*] gagalli@uchicago.edu

SUPPLEMENTARY NOTE 1: DEFECT FORMATION ENERGIES

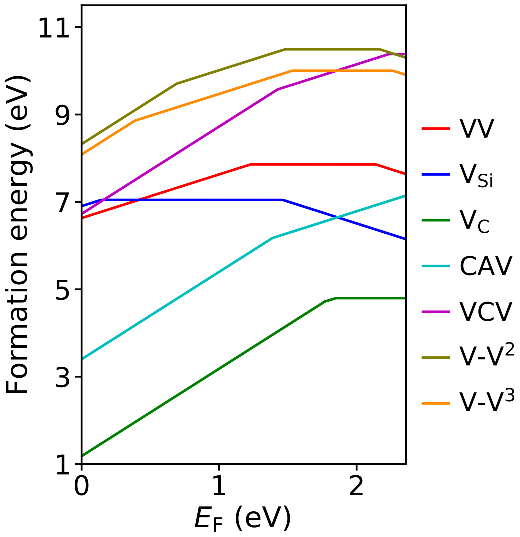

Supplementary Fig. 1. Formation energy of defects in 3C-SiC obtained under C-rich conditions, obtained using DFT and the DDH functional. The geometry of these defects can be found in Fig. 1. See Methods in the manuscript.

SUPPLEMENTARY NOTE 2: COLLECTIVE VARIABLES AND FREE ENERGY BARRIERS

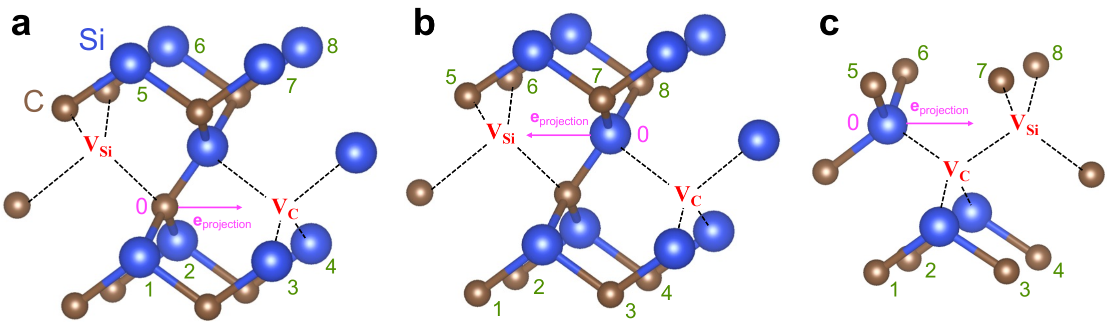

Supplementary Fig. 2. Collective variables for three paths investigated in our simulations. Number 0 denotes the moving atom; numbers 1-8 denote the gate atoms; the magenta arrows indicate the projection vectors. **a** V-V^3 → VV @ V_C path. **b** V-V^3 → VV @ V_{Si} path. **c** First step in VV migration path (see Fig. 1 in the manuscript).

We obtained the free energy surface at 1,500 K using the adaptive biasing force method. In our enhanced sampling calculations, we considered three pathways, as shown in Supplementary Fig. 2, where we highlight the moving atom, gate atoms and projection vectors used to define the collective variable (see Methods in the manuscript). For each pathway, we computed the free energy barrier G_b and entropy change ΔS from the initial to the transition state, which we denote as forward (F) direction; we then computed the G_b and ΔS from the final to the transition state, which we denote as backward (B) direction. We summarize the computed values of G_b and ΔS in Supplementary Table I.

For the three pathways studied here at 1,500 K, G_b decreases by $\sim$ (0.11, 0.38) eV, relative to the value at 0 K. Using the harmonic approximation and classical statistics (see Supplementary Note 5), we estimate ΔS in the range of $\sim$ (0.85, 2.90) k_B. In particular, in going from the stable VV configuration to the transition state, the G_b decreases by $\sim$ 0.3 eV and the corresponding ΔS is $\sim$ 2.3 k_B; the latter value is used to estimate the activation temperature (see Supplementary Note 5).

We note that the values of ΔS were computed at the PBE level of theory (ΔS^{PBE}). Using the harmonic approximation and classical statistics, ΔS is determined from phonon frequencies. Let us consider one phonon mode for simplicity, and denote the phonon frequency (ω)

at the initial (IS) and transition (TS) states as $\omega_{\rm IS}$ and $\omega_{\rm TS}$, respectively. Then, ΔS can be computed as: $\Delta S = k_{\rm B} \ln(\omega_{\rm IS}/\omega_{\rm TS})$ [1, 2]. Recent results [3] showed that the phonon frequencies obtained from PBE ($\omega^{\rm PBE}$) and DDH ($\omega^{\rm DDH}$) functionals for 4H-SiC are similar, with $\omega^{\rm DDH} \approx 1.03\ \omega^{\rm PBE}$. It is reasonable to assume that such scaling relationship holds also in the case of 3C-SiC. Since the scaling factors entering the expression of ΔS cancel out, we can easily obtain ΔS at the DDH level of theory: $\Delta S^{\rm DDH} = k_{\rm B} \ln(\omega_{\rm IS}^{\rm DDH}/\omega_{\rm TS}^{\rm DDH}) \approx k_{\rm B} \ln(\omega_{\rm IS}^{\rm PBE}/\omega_{\rm TS}^{\rm PBE})$, and we find $\Delta S^{\rm PBE} \approx \Delta S^{\rm DDH}$.

Supplementary Table I. Free energy barriers $G_{\rm b}$ (eV) at 0 K ($G_{\rm b}$ @ 0 K) and 1,500 K ($G_{\rm b}$ @ 1,500 K), free energy barrier change $\Delta G_{\rm b} = G_{\rm b}$@ 0 K $- G_{\rm b}$@ 1,500 K (eV) and entropy change $\Delta S = \Delta G_{\rm b}/T$ ($k_{\rm B}$), where $k_{\rm B}$ is the Boltzmann constant and T = 1,500 K.

$G_{\rm b}$					
Pathways	V-V^3 → VV @ V_C		V-V^3 → VV @ V_{Si}		VV migration[a]
Direction[b]	F	B	F	B	F (=B)
$G_{\rm b}$ @ 0 K	2.35	4.34	1.29	3.28	3.07
$G_{\rm b}$ @ 1,500 K	2.24	4.06	1.14	2.98	2.70
$\Delta G_{\rm b}$					
$\Delta G_{\rm b}$	0.11	0.28	0.15	0.30	0.38
ΔS					
ΔS	0.85	2.17	1.16	2.32	2.90

[a] Only the first step in VV migration path is simulated, as shown in Supplementary Fig. 2**c**.

[b] F refers to forward direction from the initial to the transition state; B refers to backward direction from the final to the transition state.

SUPPLEMENTARY NOTE 3: CALCULATION OF EFFECTIVE BARRIERS

During transformations occurring at high temperature (T), point defects may be in several charge (q) and spin (s) states different from the thermodynamically stable ones. Hence we should consider different energy barriers $E_{\rm b}(q, s)$. Moreover, the transition between different q and s states may occur at elevated T due, e.g., to vibrational effects. For these reasons, we used effective barriers $E_{\rm b,\ EFF}$ [4, 5] to describe atomic processes occurring at high T, instead of simple barriers $E_{\rm b}$.

In our NEB calculations, we first determined the most stable s state for each image at a given q; the corresponding total energies and atomic forces were then used to determine the minimum energy path and $E_{\rm b}$. Hence the final $E_{\rm b}$ obtained in this way is only a function of q, with the effect of the s degree of freedom (DOF) included implicitly. Our treatment of the spin DOFs assumes an instantaneous equilibration of spin states during defects' transformations. Although we could not estimate the timescale of s transitions via spin-orbital-coupling or spin-phonon interactions at $\sim$ 1,000 K, we found that in general considering different spin states affects only slightly the computed $E_{\rm b}$ (see Methods in the manuscript).

We computed $E_{\rm b,\ EFF}$ based on $E_{\rm b}$ and defect formation energies, considering only the charge DOF (see Computational strategy in the manuscript). We assumed the charge state q to be preserved during defect transformations, due to the short lifetime of barrier crossing over the transition state, and we considered q transitions at the initial and final states of a given path.

In the case of the dissociation of complex defects, involving multiple steps, $E_{\rm b,\ EFF}$ was estimated from the binding energy and diffusion barriers. For instance, $E_{\rm b,\ EFF}$ for the CAV dissociation process was obtained as the sum of the binding energy (C_{Si} & V_C) and the $E_{\rm b,\ EFF}$ of V_C migration. We computed the CAV migration barrier from the formation energy difference ($\Delta E_{\rm f} = E_{\rm f}(V_{Si}) - E_{\rm f}(\rm CAV)$), the V_{Si} migration barrier and the $V_{Si} \rightarrow$ CAV barrier. Specifically, if $\Delta E_{\rm f} \geq 0$ eV, i.e. the initial state CAV is more stable than the intermediate state V_{Si}:

$$E_{\rm b,\ EFF}(\text{CAV migration}) = \Delta E_{\rm f} + \max\big\{E_{\rm b,\ EFF}(V_{Si}\ \text{migration}), E_{\rm b,\ EFF}(V_{Si} \rightarrow \text{CAV})\big\} \quad (1)$$

; if $\Delta E_\mathrm{f} < 0$ eV, the intermediate state V_{Si} is more stable:

$$E_{\mathrm{b,\ EFF}}(\mathrm{CAV\ migration}) = \max\left\{E_{\mathrm{b,\ EFF}}(\mathrm{V_{Si}\ migration}), E_{\mathrm{b,\ EFF}}(\mathrm{V_{Si}} \rightarrow \mathrm{CAV})\right\} \quad (2)$$

To obtain an accurate value of $E_{\mathrm{b,\ EFF}}$, the charge state equilibration of defects , i.e. the transition between different charge states, should be fast compared to the transformation of defects into different configurations. We estimated that the charge state equilibration is indeed fast at high T (see Supplementary Note 4 below).

SUPPLEMENTARY NOTE 4: TIMESCALE OF CHARGE STATE EQUILIBRATION

We obtained the timescale of the charge state equilibration of defects by estimating the carrier capture and emission rates. Under equilibrium conditions, the electron (hole) capture rate k_e (k_h) and the electron (hole) emission rate g_e (g_h) [5, 6] can be estimated as:

$$k_\mathrm{e}(T) \approx \sigma\langle v\rangle\gamma N \exp(-\frac{E_\mathrm{C}-E_\mathrm{F}}{k_\mathrm{B}T}) \tag{3}$$

$$k_\mathrm{h}(T) \approx \sigma\langle v\rangle\gamma N \exp(\frac{E_\mathrm{V}-E_\mathrm{F}}{k_\mathrm{B}T}) \tag{4}$$

$$g_\mathrm{e}(T) \approx \sigma\langle v\rangle\gamma N \exp(-\frac{E_\mathrm{C}-E_\mathrm{T}}{k_\mathrm{B}T}) \tag{5}$$

$$g_\mathrm{h}(T) \approx \sigma\langle v\rangle\gamma N \exp(\frac{E_\mathrm{V}-E_\mathrm{T}}{k_\mathrm{B}T}) \tag{6}$$

where σ is the capture cross section; $\langle v\rangle$ is the average thermal velocity of carriers; γ is the degeneracy factor; N is the effective density of states (DOS); E_V is the valence band maximum energy; E_C is the conduction band minimum energy; E_F is the Fermi level; E_T is the defect level; k_B is the Boltzmann constant.

Here, we estimated the k_e and k_h (g_e and g_h) at 1,000 K, as a function of E_F (E_T) for 3C- and 4H-SiC. Given $N(T) \sim T^{3/2}$ [7, 8], we estimated $N(1,000\ \mathrm{K}) \sim N(300\ \mathrm{K}) \times (10/3)^{3/2}$. $N(300\ \mathrm{K})$ was measured experimentally [7]. Given $\langle v\rangle(T) \sim (3k_\mathrm{B}T/m_0)^{0.5}$, where m_0 is the mass of stationary electron; we estimated $\langle v\rangle(1,000\ \mathrm{K}) \sim 2\times10^7$ cm s^{-1}. Based on experiments, for most of deep levels in SiC, σ are in the range of (10^{-17}, 10^{-14}) cm^2 [9–12]. We assumed $\gamma = 1$. We obtained the band gap of SiC at 1,000 K using its temperature dependence reported in Ref. [7]. Our results are presented in Supplementary Fig. 3. We find that both carrier capture and emission rates for defects in 3C-SiC are on the order of $<$ 1 s (Supplementary Fig. 3**a**). However, for 4H-SiC (Supplementary Fig. 3**b**), the timescale could be longer due to a larger band gap. In particular, when E_F (E_T) is close to the band edge, the timescale, k_e^{-1} or k_h^{-1} (g_e^{-1} or g_h^{-1}), exceeds 1 s. However, since the E_F would approach the mid-gap (see Supplementary Figs. 4 and 5) at high T, we expect that both the electron and hole capture processes will be fast with k_e^{-1} and $k_\mathrm{h}^{-1} < 1$ s. Note that here we have ignored the T dependence of the capture cross section (σ). For instance, assuming multiphonon processes, $\sigma \propto \exp(-\Delta E/k_\mathrm{B}T)$ where ΔE is an energy barrier [6, 13]. Thus, the results presented in Supplementary Fig. 3 may be overestimated. Even in the case where

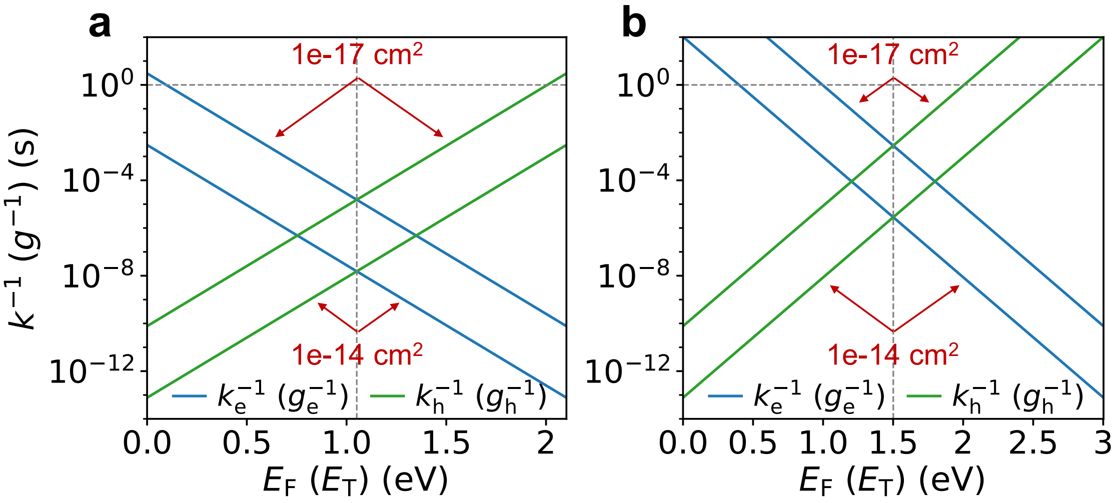

Supplementary Fig. 3. Estimated timescale for carrier capture (k^{-1}) and emission (g^{-1}) processes at 1,000 K. We computed the timescales for the electron (k_e^{-1}) and hole (k_h^{-1}) capture as a function of Fermi level (E_F). We computed the timescales for the electron (g_e^{-1}) and hole (g_h^{-1}) emission as a function of defect level (E_T). The E_F and E_T are referred to the top of the valence band. We show results for 3C-SiC (**a**) and 4H-SiC (**b**). The values of mid-gap and timescale of 1 s are indicated by dashed lines. We used the band gap computed at 1,000 K. The used capture cross sections (σ) are specified in red text. See text for computational details.

the results represent an overestimate, the timescale for defect transformations at 1,000 K with barrier $\sim$ 3 eV is estimated to be $\sim$ 10 s, i.e. an order of magnitude longer. Therefore, it is reasonable to assume fast charge state equilibrations, justifying our use of effective barrier $E_{\text{b, EFF}}$.

SUPPLEMENTARY NOTE 5: CALCULATIONS OF THE ACTIVATION TEMPERATURE

According to the harmonic transition state theory [1, 2], the jump frequency $\mathit{\Gamma}$ can be calculated as:

$$\mathit{\Gamma} = \mathit{\Gamma}_0 \exp(-G_{\mathrm{b}}/k_{\mathrm{B}}T) \tag{7}$$

where $\mathit{\Gamma}_0$ is the attempt frequency and G_{b} the free energy barrier of a given process.

During defect transformations, we assumed the system volume to be constant, hence:

$$G_{\mathrm{b}} = \Delta U - T\Delta S \tag{8}$$

where ΔU is the change in internal energy from the initial to the transition state of a given path; ΔS is the change in entropy from the initial to the transition state of the path. Note that we computed ΔS for three paths only, due to the computational cost, and found values varying within $\sim$ (0.85, 2.90) k_{B} (see Supplementary Note 2). Based on the harmonic approximation and classical statistics, the change in kinetic energy is 0 eV, and the change in potential energy is E_{b} (barrier at 0 K), enforced by the equi-partition theorem. In addition, ΔS is constant, as determined by calculations of phonon frequencies [1, 2]. Therefore, we obtain:

$$\mathit{\Gamma} \approx \mathit{\Gamma}_0 \exp(\frac{\Delta S}{k_{\mathrm{B}}}) \exp(-E_{\mathrm{b}}/k_{\mathrm{B}}T) \tag{9}$$

The activation temperature T_a is defined as the T above which a process is thermally activated. Based on Supplementary Equation (9), T_a can be written as:

$$T_{\mathrm{a}} = \left[k_{\mathrm{B}} \ln(\mathit{\Gamma}_0 \exp(\frac{\Delta S}{k_{\mathrm{B}}})/\mathit{\Gamma})\right]^{-1} \times E_{\mathrm{b,\ EFF}} \tag{10}$$

Similar to previous studies, we approximated $\mathit{\Gamma}_0$ to be 1.6×10^{13} Hz [14]; jump frequency $\mathit{\Gamma}$ to be 0.1 Hz [15, 16]; ΔS to be 2.3 k_{B}. This value corresponds to our estimate of the ΔS from the stable VV configuration to the transition state (see Supplementary Note 2). We obtained a prefactor (inverse of the quantity within square brackets in Supplementary Equation (10)) of 331 K eV^{-1}. A simple sensitivity analysis shows that such prefactor is relatively insensitive to the choice of $\mathit{\Gamma}$ and ΔS. For example, by varying $\mathit{\Gamma}$ in the range of (0.01, 1) Hz (with all other parameters fixed), the prefactor changes by $< \sim 7$ %; by varying ΔS in the range of (0.85, 2.90) k_{B}, the prefactor changes by $< \sim 4$ %.

We systematically investigated the thermal expansion and entropic effects on computed energy barriers. We found that the lattice expansion, 4.416 Å at 1,500 K vs. 4.36 Å at 0 K [17], leads to a minor change of E_{b} (which is on the order of several eV) of approximately $\sim \pm$ 0.1 eV for most processes, with the exception of small carbon-clusters' formation for which differences in energy barriers are $\sim \pm$ 0.3 eV. We found that due to entropic effects, our computed free energy barriers are lowered by $\sim$ (0.11, 0.38) eV at 1,500 K, relative to those obtained at 0 K (see Supplementary Note 2), consistent with estimates based on the harmonic approximation [14]. As a result, we estimate that the variation of T_{a} due to thermal expansion and entropic effects is less than 10 % .

SUPPLEMENTARY NOTE 6: CALCULATIONS OF THE FERMI LEVEL

After irradiation or implantation of SiC samples, multiple defects can be created including interstitials, antisites, substitutionals and vacancies. In order to obtain an accurate value of E_F we need to consider both external doping and the charge state of the defects created in the sample.

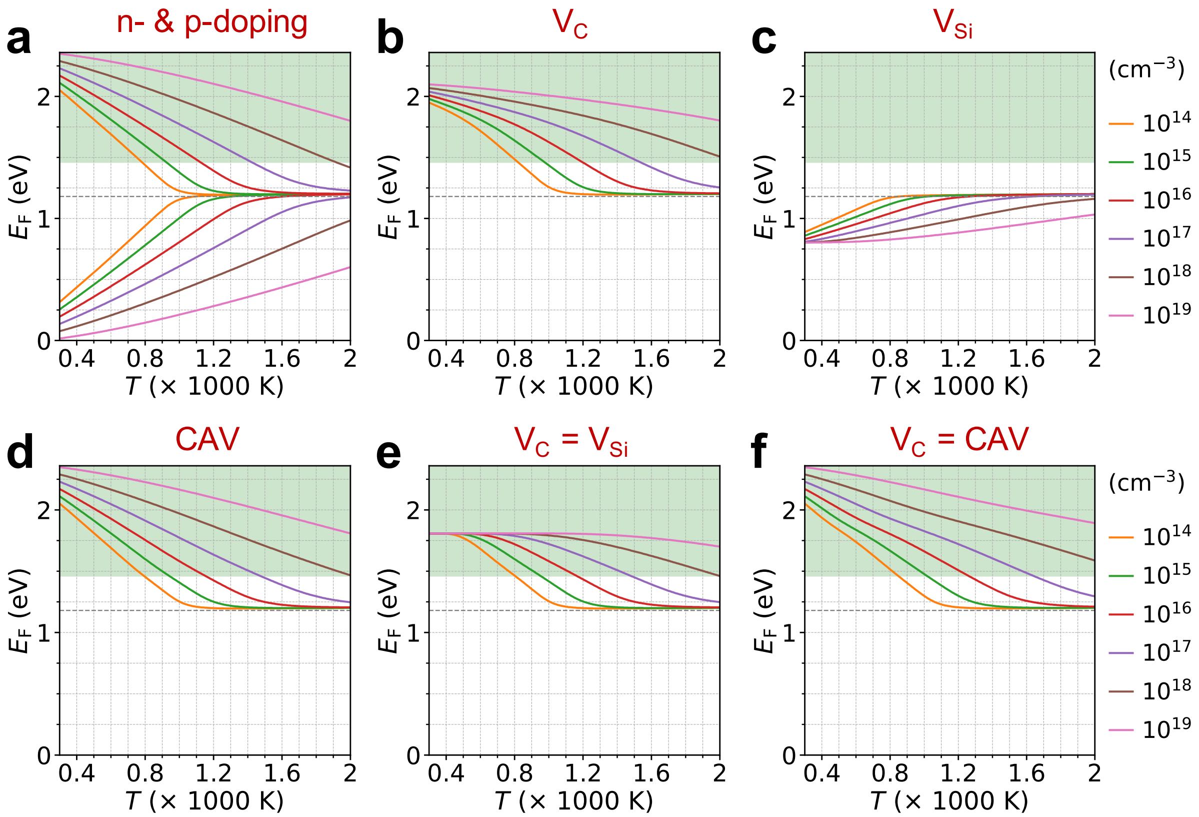

Supplementary Fig. 4. Fermi level (E_F) as a function of temperature (T) in 3C-SiC. The Fermi level is referred to the top of the valence band. We consider the doping or defects density in the range of $(10^{14}, 10^{19})$ cm^{-3}. **a** Presence of n- or p-doping only. **b** Presence of carbon vacancy (V_C) only. **c** Presence of silicon vacancy (V_{Si}) only. **d** Presence of carbon antisite vacancy (CAV) only. **e** Presence of V_C and V_{Si} of the same amount. **f** Presence of V_C and CAV of the same amount. The value of mid-gap for 3C-SiC is indicated by a grey dashed line; the green-region for $E_F > 1.46$ eV indicates the suitable conditions for the VV creation.

In this study, we took into account several vacancies: V_C, V_{Si} and CAV, which are relevant

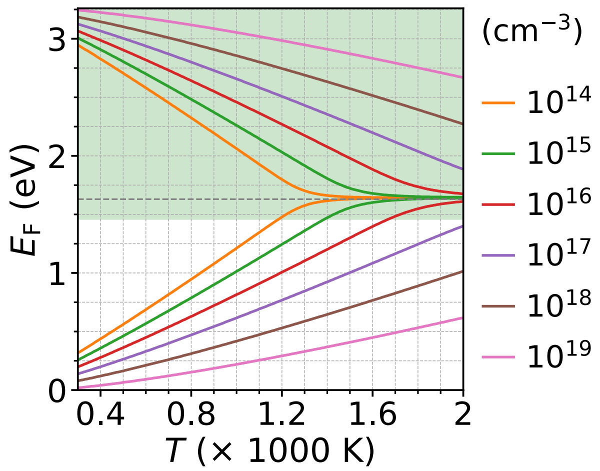

Supplementary Fig. 5. Fermi level (E_{F}) as a function of temperature (T) in 4H-SiC. The Fermi level is referred to the top of the valence band. We consider the n- or p-doping density in the range of (10^{14}, 10^{19}) cm^{-3}. The value of mid-gap for 4H-SiC is indicated by a grey dashed line; the green-region for $E_{\mathrm{F}} > 1.46$ eV indicates the suitable conditions for the VV creation.

to the VV creation processes. We determined E_{F} using the following equations [8, 18, 19]:

$$\begin{cases} \text{a)} \quad n_0 p_0 = N_{\mathrm{C}} N_{\mathrm{V}} \exp(-E_{\mathrm{g}}/k_{\mathrm{B}}T) \\ \text{b)} \quad n_0 + N_{\mathrm{a}} = p_0 + N_{\mathrm{d}} + \sum_{\mathrm{X}\in(\mathrm{V_C},\mathrm{V_{Si}},\mathrm{CAV})} \sum_q q \times N(\mathrm{X}^q) \\ \text{c)} \quad N(\mathrm{X}^q) \propto N_{\mathrm{X}} g_{\mathrm{X}^q} \exp(-E_{\mathrm{f}}(\mathrm{X}^q)/k_{\mathrm{B}}T) \\ \text{d)} \quad E_{\mathrm{F}} - E_{\mathrm{V}} = k_{\mathrm{B}}T \ln(N_{\mathrm{V}}/p_0) \end{cases} \tag{11}$$

where n_0 is the electron density; p_0 is the hole density; N_{C} is the effective conduction band DOS; N_{V} is the effective valence band DOS; E_{g} is the band gap; N_{a} is the acceptor density; N_{d} is the donor density; X^q stands for defect X in charge state q; $N(\mathrm{X}^q)$ is the density of X^q; N_{X} is the total density of defect X; g is the degeneracy factor; taken as 1; E_{f} is the defect formation energy (see Supplementary Note 1); E_{V} is the valence band maximum (VBM) energy.

Supplementary Equation (11)b expresses the charge neutrality condition, incorporating the effects of defects. These equations need to be solved self-consistently. Here, we performed a line-search by step-wisely increasing E_{F} from VBM to the conduction band minimum; we determined E_{F} as the value, which makes Supplementary Equation (11)b satisfied with a minimal error. The electronic properties of SiC were obtained from the Appendix C of the

book [7]. For simplicity, we ignored the T dependence of these parameters: 1) E_{g} at 300 K was used; 2) E_{f} diagram at 0 K was used (Supplementary Fig. 1). We deduced the DOS effective masses based on the measured N_{C} and N_{V} at 300 K, which were then used to compute N_{C} and N_{V} at various T. Some of our results are shown in Supplementary Figs. 4 and 5. We note our E_{F} may be over-estimated, since band gap decreases at high T.

Overall, the calculation of E_{F} here should be taken as a qualitative estimate, as we: 1) ignored the T dependence of electronic properties, e.g. band gap of SiC; 2) ignored the effects of other defects, in addition to $\mathrm{V_C}$, $\mathrm{V_{Si}}$ and CAV. More accurate treatment is beyond the scope of this work.

SUPPLEMENTARY NOTE 7: COMPUTED ACTIVATION TEMPERATURES USING ENERGY BARRIERS

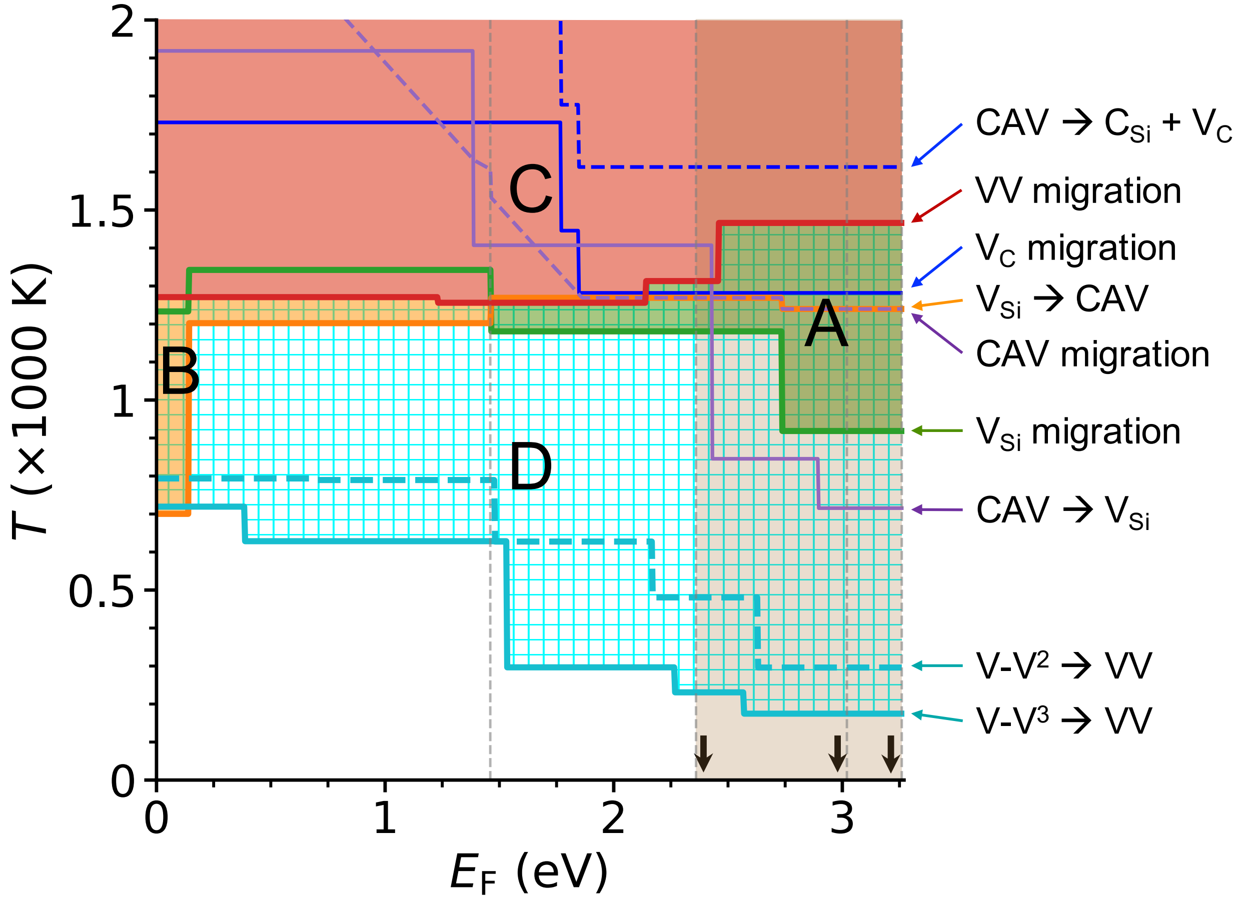

Supplementary Fig. 6. Computed activation temperature as a function of the Fermi level (E_F). The Fermi level is referred to the top of the valence band. This plot is the same as Fig. 3 in the manuscript, except that here we used energy barriers (E_b) determined from the most stable charge-state at a given E_F while effective barriers ($E_{b,\ EFF}$) were used in Fig. 3 (see text and caption of Fig. 3).

SUPPLEMENTARY REFERENCES

[1] G. H. Vineyard, Frequency factors and isotope effects in solid state rate processes, J. Phys. Chem. Solids **3**, 121 (1957).

[2] J. Li, The mechanics and physics of defect nucleation, MRS Bull. **32**, 151 (2007).

[3] Y. Jin, M. Govoni, G. Wolfowicz, S. E. Sullivan, F. J. Heremans, D. D. Awschalom, and G. Galli, Photoluminescence spectra of point defects in semiconductors: Validation of first-principles calculations, Phys. Rev. Mater. **5**, 084603 (2021).

[4] U. Gerstmann, E. Rauls, and H. Overhof, Annealing of vacancy-related defects in semi-insulating SiC, Phys. Rev. B **70**, 201204 (2004).

[5] F. Bruneval and G. Roma, Energetics and metastability of the silicon vacancy in cubic SiC, Phys. Rev. B **83**, 144116 (2011).

[6] J. Bourgoin and M. Lannoo, *Point defects in Semiconductors II: Experimental aspects*, Vol. 35 (Springer-Verlag, Berlin, 1983).

[7] T. Kimoto and J. A. Cooper, *Fundamentals of silicon carbide technology: growth, characterization, devices and applications* (John Wiley & Sons, 2014).

[8] D. A. Neamen, *Semiconductor Physics and Devices: Basic Principles* (McGraw-Hill, 2012).

[9] H. Nakane, M. Kato, Y. Ohkouchi, X. T. Trinh, I. G. Ivanov, T. Ohshima, and N. T. Son, Deep levels related to the carbon antisite–vacancy pair in 4H-SiC, J. Appl. Phys. **130**, 065703 (2021).

[10] P. Hazdra and J. Vobeckỳ, Radiation defects created in n-Type 4H-SiC by electron irradiation in the Energy Range of 1–10 Mev, Phys. Stat. Solidi A **216**, 1900312 (2019).

[11] K. Danno and T. Kimoto, Deep level transient spectroscopy on as-grown and electron-irradiated p-type 4H-SiC epilayers, J. Appl. Phys. **101**, 103704 (2007).

[12] S. M. Tunhuma, M. Diale, M. J. Legodi, J. M. Nel, T. Thabete, and F. D. Auret, Defects induced by solid state reactions at the tungsten-silicon carbide interface, J Appl. Phys. **123**, 161565 (2018).

[13] I. D. Booker, E. Janzén, N. T. Son, J. Hassan, P. Stenberg, and E. Sveinbjörnsson, Donor and double-donor transitions of the carbon vacancy related $EH_{6/7}$ deep level in 4H-SiC, J. Appl. Phys. **119**, 235703 (2016).

[14] E. Rauls, T. Frauenheim, A. Gali, and P. Deák, Theoretical study of vacancy diffusion and

vacancy-assisted clustering of antisites in SiC, Phys. Rev. B **68**, 155208 (2003).

[15] A. Kyrtsos, M. Matsubara, and E. Bellotti, Migration mechanisms and diffusion barriers of vacancies in Ga_2O_3, Phys. Rev. B **95**, 245202 (2017).

[16] Y. K. Frodason, C. Zimmermann, E. F. Verhoeven, P. M. Weiser, L. Vines, and J. B. Varley, Multistability of isolated and hydrogenated Ga–O divacancies in β- Ga_2O_3, Phys. Rev. Mater. **5**, 025402 (2021).

[17] P. Haas, F. Tran, and P. Blaha, Calculation of the lattice constant of solids with semilocal functionals, Phys. Rev. B **79**, 085104 (2009).

[18] J. Ma, S.-H. Wei, T. Gessert, and K. K. Chin, Carrier density and compensation in semiconductors with multiple dopants and multiple transition energy levels: Case of Cu impurities in CdTe, Phys. Rev. B **83**, 245207 (2011).

[19] J. Buckeridge, Equilibrium point defect and charge carrier concentrations in a material determined through calculation of the self-consistent Fermi energy, Comput. Phys. Commun. **244**, 329 (2019).
